# Phasic Stimulation of Midbrain Dopamine Neuron Activity Reduces Salt Consumption

**DOI:** 10.1523/ENEURO.0064-18.2018

**Published:** 2018-05-15

**Authors:** Eleanor C. Sandhu, Anushka B. P. Fernando, Elaine E. Irvine, Kyoko Tossell, Michelle Kokkinou, Justyna Glegola, Mark A. Smith, Oliver D. Howes, Dominic J. Withers, Mark A. Ungless

**Affiliations:** 1 MRC London Institute of Medical Sciences (LMS), London W12 0NN, United Kingdom; 2 Institute of Clinical Sciences (ICS), Faculty of Medicine, Imperial College London, London W12 0NN, United Kingdom; 3 Department of Psychosis Studies, Institute of Psychiatry, Psychology & Neuroscience, Kings College London, London SE5 8AF, United Kingdom

**Keywords:** appetite, dopamine, salt, VTA

## Abstract

Salt intake is an essential dietary requirement, but excessive consumption is implicated in hypertension and associated conditions. Little is known about the neural circuit mechanisms that control motivation to consume salt, although the midbrain dopamine system, which plays a key role in other reward-related behaviors, has been implicated. We, therefore, examined the effects on salt consumption of either optogenetic excitation or chemogenetic inhibition of ventral tegmental area (VTA) dopamine neurons in male mice. Strikingly, optogenetic excitation of dopamine neurons decreased salt intake in a rapid and reversible manner, despite a strong salt appetite. Importantly, optogenetic excitation was not aversive, did not induce hyperactivity, and did not alter salt concentration preferences in a need-free state. In addition, we found that chemogenetic inhibition of dopamine neurons had no effect on salt intake. Lastly, optogenetic excitation of dopamine neurons reduced consumption of sucrose following an overnight fast, suggesting a more general role of VTA dopamine neuron excitation in organizing motivated behaviors.

## Significance Statement

Although it is well-established that midbrain dopamine neurons are involved in many types of reward-related behaviors, little is known about their role in salt intake under conditions where salt is appetitive (i.e., during salt depletion). Here, we show that optogenetic excitation of midbrain dopamine neurons can decrease salt intake. Importantly, this stimulation protocol did not affect salt concentration preferences. Furthermore, we find that this stimulation protocol can also reduce sucrose intake following an overnight fast, suggesting a broader role for dopamine neuron activity in regulating nutrient intake, which compliments findings from previous lesion- and pharmacological-based studies.

## Introduction

Dietary sodium intake is essential to the regulation of fluid and electrolytes within the body. Indeed, chronic salt depletion, through diet or the use of low sodium dialysate during dialysis, has been associated with increased mortality (Alderman and Cohen, 2012). Moreover, in certain patient groups (for example the elderly and during diarrheal illness) the physiologic ability to respond to salt depletion is impaired due to medication or illness. This is significant since hyponatremia has profound multi-organ consequences which may be fatal. It is therefore essential to maintain total body salt homeostasis. This is normally achieved through physiologic control of loss and intake. Accordingly, a strong sodium appetite occurs in response to low levels of sodium in the body resulting in consumption of high salt foods (Richter, 1956; Denton, 1982). However, excessive salt intake, beyond metabolic need, leads to increased blood pressure and the risk of both cardiovascular disease and obesity (Ma et al., 2015). There are major public health initiatives to reduce salt intake (World Action on Salt and Health; http://www.worldactiononsalt.com). Despite these, there has been little evidence of a reduction in salt consumption at a population level (McCarron et al., 2013; Asayama et al., 2014). There is, therefore, a pressing need to understand better the mechanisms through which salt consumption is mediated and to develop therapeutic interventions that could regulate intake.

One useful framework for understanding the neural basis of salt appetite proposes that it is regulated by three distinct neural components (Geerling and Loewy, 2008). First, salt depletion is detected by subfornical organ neurons and nucleus of the solitary tract (NTS) neurons expressing the enzyme 11β-hydroxysteroid dehydrogenase type 2 (HSD2). These neurons are excited by salt depletion and when stimulated can drive salt appetite (Geerling and Loewy, 2008; Nation et al., 2016; Jarvie and Palmiter, 2017). Second, gustatory signals transmit information about the detection of salt via non-HSD2 expressing NTS neurons (Geerling and Loewy, 2008). Third, these two signals are integrated in forebrain sites to drive motivated behavior to consume salt (Geerling and Loewy, 2008). These forebrain sites include the mesocorticolimbic dopamine system, which plays a key role in processing information about other types of reward (Wise, 2006). There is some evidence linking the reinforcing properties of salt intake to the dopamine system. For example, dopamine type 2 receptor (D2R) antagonists reduce sham drinking of sodium chloride (NaCl) where fluid empties through a gastric fistula minimizing post-ingestive inhibitory signals when compared to normal drinking of NaCl (Roitman et al., 1997). Furthermore, salt depletion results in an increase in dopamine release in the nucleus accumbens on unconditioned presentation of NaCl, which is not seen when the animal is salt replete suggesting salt appetite positively modulates dopamine signaling (Cone et al., 2016). Taken together these findings suggest that during salt appetite, salt becomes appetitive and engages neural circuits (particularly the dopamine system) that are involved in mediating appetitive behavior toward other types of reward. However, it is not well understood how changes in dopamine neuron activity affect salt intake. Interestingly, excitation of dopamine neurons has recently been shown to suppress sucrose drinking and feeding behavior (Mikhailova et al., 2016; Boekhoudt et al., 2017). We, therefore, hypothesized that under conditions of salt appetite, excitation of dopamine neurons would suppress salt intake.

## Materials and Methods

### Animals

Mice were housed in cages of two to four animals and maintained on a 12/12 h light/dark cycle. Before any changes in diet, food and water were available *ad libitum*. Male C57BL6 mice, 16–18 weeks, (Charles River; IMSR catalog #CRL:27, RRID:IMSR_CRL:27) were used in non-optogenetic studies. For the optogenetic studies, male DAT-iCre heterozygous mice (DATcre+) and wild-type litter mates (DATcre-) on a C57BL/6 background were used (IMSR catalog #EM:01738, RRID:IMSR_EM:01738; Turiault et al., 2007). Animal husbandry and experimental procedures were undertaken in accordance with the United Kingdom Animal (Scientific Procedures) Act of 1986.

### Virus

The DIO-ChR2-mCherry construct was kindly gifted by the Deisseroth Lab and the viral particles were produced by Vector Biolab, Philadelphia. Concentrations varied minimally with batches of virus across experiments, they ranged from 2.7 to 5.8 × 10^–13^ GC/ml but were diluted down to 2.0 × 10^–12^ GC/ml. Designer receptors exclusively activated by designer drugs (DREADD) construct of human muscarinic acetylcholine receptor M4 fused to mCherry (hM4Di-mCherry) was constructed according to Nawaratne et al. (2008) and cloned into pAAV-Eifla-DIO-WPRE vectors. DREADD construct of human muscarinic acetylcholine receptor M3 fused to mCherry (hM3Dq-mCherry) was obtained from Prof. Graeme Milligan, University of Glasgow, and was inserted into pAAV-Eifla-DIO-WPRE vectors (gift from Deisseroth Lab, Stanford, http://web.stanford.edu/group/dlab/optogenetics/sequence_info.html). The vectors were packaged in adeno-associated virus (AAV) serotype 2/1 vector consisting of the AAV2 ITR genomes and the AAV1 serotype capsid gene, titer 2.1 × 10^–12^ GC/ml (Vector Biolabs).

### Surgery

Ten to twelve-week-old mice were anaesthetized with isoflurane (5% for induction; 1–2% for maintenance) and placed into a Kopf stereotaxic frame (Bilaney Consultants). A total of 0.25% bupivacaine was injected subcutaneously beneath the scalp before an incision was made down the midline. For AAV injections, holes were drilled bilaterally to target the ventral tegmental area (VTA) using the coordinates AP -3.45 mm, ML ±0.4 mm, and DV -5.05 mm. For optogenetic stimulation studies, to accommodate implantable fibers a 10° angle was used and coordinates were ML ±1.3 mm, DV −4.89 mm (injection), and −4.44 mm (optical fiber). A total of 0.5 µl of AAV was injected, bilaterally into the VTA using a 33-gauge metal needle and a 5-µl Hamilton glass syringe at a rate of 0.15 µl/min. The needle was left for 5 min after injection before being slowly removed. Following this, implantable optical fibers (constructed according to Sparta et al., 2011) were placed bilaterally, via the same craniotomies. Two screws were placed dorsal to lambda and anterior to bregma to anchor a dental cement cap. Mice recovered from anesthesia in a heated chamber, were group housed, and left to recover for at least two weeks before handling and habituation. Microscopic inspection, under light anesthesia, of a test cohort showed no damage to the ferrules by littermates after two weeks.

### Handling and habituation

Mice were handled for two weeks and habituated to the testing jellies and apparatus for one week before the test session.

### Induction of appetite

For the salt appetite experiments, an acute sodium depletion protocol was used. Briefly, two weeks before testing, mice were switched to low sodium diet (RM < 0.025% Na; Special Diets Services) with access to NaCl, via a glass dish, containing 0.4% agar jelly and 0.75 M NaCl in their home cage. Two days before the test session, mice were injected intraperitoneally with furosemide (20 mg/kg; Hameln Pharmaceuticals), their cages changed and the NaCl jellies were replaced with 0.4% agar jellies. This was repeated for a second day, followed by the test day. Saline controls followed the same protocol but were maintained on normal chow with NaCl jellies throughout and were injected with vehicle (NaCl; 0.9% 2 ml/kg; Animalcare Ltd) for 2 d before the test session. The experimenter was blind to group allocation and setup. A fasted state was achieved with removal of all food at 4 P.M. before the test day, although water remained *ad libitum*. This overnight-fasted state was used in experiments testing sucrose appetite and in the need-free sodium preference experiment.

### Test procedure

Mice were tethered to the laser via patchcords and placed in their designated testing chamber (base of Allentown XJ cage, 19.37 × 38.13 × 13.03 cm) with a paper liner. Following 10 min of habituation, laser stimulation was started. Five minutes into the stimulation protocol, three jellies of three different concentrations were placed at one end of the chamber. The order of jellies was systematically randomized and was kept constant throughout habituation and testing for each mouse. Mice were allowed to freely consume the jellies for the 30-min test session. The jellies were removed every 10 min, weighed and then returned to the chamber. For chemogenetic experiments the mice were injected with clozapine-*N*-oxide (CNO; 0.1 mg/kg; i.p.) and placed in their testing chamber. Jellies were placed in the test chamber after 30 min, and the test session began.

### Optical stimulation

For optical stimulation studies, implantable optic fibers were attached to a 1 × 2 intensity division fiber-optic rotary joint (Doric Lenses Inc.) using patchcords (Doric Lenses Inc) via a zirconia sleeve. An insulated optical fiber connected the rotary joint to a 473-nm laser source (CrystaLaser and Vortran Laser Technology Inc.). Light output was adjusted by measuring the light output from the tip of an implantable optical fiber using an optical power meter, aiming for a power of 2–3 mW from the tip of the implanted ferule. A phasic illumination pattern was used in experiments with channelrhodopsin (ChR2). This consisted of eight pulses of 5-ms pulse width, spaced 37 ms apart, every 5 s, similar to previously published optogenetic stimulation studies (Adamantidis et al., 2011; Tye et al., 2013).

### Immunohistochemistry

Mice were anaesthetized with isoflurane (5%) and then 0.08-ml Euthatal 100 mg/ml intraperitoneally and perfused transcardially with ∼30 ml of 0.01 M PBS followed by 100 ml of PBS containing 4% paraformaldehyde (PFA) at 4°C. The brain was immediately removed and postfixed in 4% PFA for 2 h. Subsequently the brains were cryoprotected in 30% sucrose in PBS, embedded in optimal cutting temperature (OCT) medium and frozen in isopentane at −50°C. Brains were stored at −80°C until they were coronally sectioned at 70 μm on a cryostat (Leica CM1800, Leica Microsystems). Free floating sections were washed four times in PBS for 5 min, then incubated with 6% normal donkey serum in 0.2% Triton X-100 in PBS (PBS-Tx). Sections were then incubated simultaneously with primary antibodies, in 2% normal donkey serum in PBS-Tx, at 4°C as follows: chicken anti-tyrosine hydroxylase (1:1000; Abcam catalog #ab76442, RRID:AB_1524535) for a minimum of 24 h, rabbit anti-cFos (1:20,000; Millipore catalog #PC38, RRID:AB_2106755) for a minimum of 74 h. Sections were then washed four times in PBS-Tx for 5 min and then incubated with the appropriate secondary antibodies: Alexa Fluor 488 donkey anti-rabbit (1:1000; Thermo Fisher Scientific catalog #R37118, RRID:AB_2556546) and Alexa Fluor 633 goat anti-chicken (1:1000; Thermo Fisher Scientific catalog #A-21103, RRID:AB_2535756) or Alexa Fluor 488 goat anti-chicken (1:1000; Thermo Fisher Scientific catalog #A-11039, RRID:AB_2534096) alone for 2 h at room temperature or 24 h at 4°C. Sections were then rinsed for 5 min, first in PBS-Tx three times then PBS two times before being mounted in Vectorshield Mounting Medium (Vector Laboratories). Confocal laser scanning microscopy was performed using a Leica SP confocal microscope. Images were taken at a resolution of 1024 × 1024 and processed using Leica Confocal Software (Leica Microsystems), Adobe Photoshop CS3 (Adobe Systems) and ImageJ. Anatomic localization of optical fibers was assessed by examining the tracts in combination with immunolabelling for tyrosine hydroxylase to identify VTA dopamine neurons.

### cFos immunostaining

Seven male mice underwent unilateral surgery. Following two weeks of recovery, mice were handled daily for two weeks and followed the standard habituation protocol. On the test day, mice were tethered to the laser via patchcords and placed in their designated testing chamber with a paper liner. Following a 15-min habituation period, 30 min of phasic stimulation started. The mice were left for 30 min, then anaesthetized, transcardially perfused, the brain removed and sectioned at 70 μm on a cryostat. Sections were processed as above. Three areas were selected in the VTA corresponding to medial, ventral, and dorsolateral regions. TH+ve cells were identified and then checked for cFos. Following cFos analysis, mCherry expression was checked in selected brain slices.

### Conditioned place preference (CPP)

Following surgery, mice were housed in pairs or trios. They were handled and scruffed for a week before the test day. A biased CPP was then performed based on Tsai et al. (2009) with 4 d of conditioning. A three compartment CPP setup was used (Med Assoc. Inc.). The white and black compartments were scented with lemon and ethanol respectively. On day 1, mice were placed in the central gray compartment for 2 min, and gates were then opened allowing free exploration of all three compartments for 15 min. The time spent in each area was recorded and their preferred chamber was noted. On the first day of conditioning (day 2), the mice were placed in one compartment and the following day (day 3) the other compartment for 30 min. They were tethered in both compartments but only stimulated in the compartment they showed least preference for on day 1. Laser power output was 2 mW from the tip. Conditioning was continued for days 4 and 5. On day 6, the mice were tested for preference. As for day 1, they were placed in the central gray compartment for 2 min, gates opened, and then allowed to explore for 15 min without stimulation. Time spent in each compartment on day 1 and day 6 was analyzed to assess whether the mice preferred the compartment where they had received stimulation. Locomotor activity on the conditioning days was analyzed to assess whether stimulation increased activity.

### Open field activity

Each mouse was tested in a custom-made wooden open field arena 45 × 45 cm with 30 cm walls. Mice were habituated to the arena for 20 min and then injected intraperitoneally with either CNO (0.1 mg/kg) or saline and immediately placed back in the arena for a 60-min test session. hM3Dq-expressing mice were previously injected with different CNO doses (ranging from 0.1 to 0.5 mg/kg) or saline. Drug allocation (saline vs CNO) was randomized before each dose tested (data not shown). The groups were then re-randomized and injected with a dose of 0.05-mg/kg CNO or saline. Their activity was recorded using a video camera suspended above the arena that interfaced with a computerized tracking system (Ethovision XT, Noldus). Total distance traveled was recorded in 1-min bins and analyzed in 5-min bins.

### In vitro electrophysiology

Ten- to twelve-week-old male mice (DATcre+ for optogenetic and chemogenetic validation experiments; C57BL6 for salt-deprivation and fasting experiments) were anaesthetized by Euthatal following isoflurane. The brain was removed by decapitation following a quick transcardial perfusion with ice-cold artificial CSF (aCSF; 120 mM NaCl, 3.5 mM KCl, 1.25 mM NaH_2_PO_4_, 25 mM NaHCO_3_, 10 mM glucose, 1 mM MgCl_2_, and 2 mM CaCl_2_) fully equilibrated with carbogen gas (95% O_2_ and 5% CO_2_).Two or three horizontal brain slices (190-μm thickness) encompassing the VTA were obtained using a vibratome (Leica VT1000S; Leica Microsystems) and were incubated for 15 min in carbogenated NMDG-HEPES recovery solution (93 mM NMDG, 2.5 mM KCl, 1.2 mM NaH_2_PO_4_, 30 mM NaHCO_3_, 20 mM HEPES, 25 mM glucose, 5 mM sodium ascorbate, 2 mM thiourea, 3 mM soduim pyrurate, 10 mM MgSO_4_, 0.5 mM CaCl_2_; pH 7.3, 300 mOsm, 33°C; Zhao et al., 2011), and transferred back to aCSF. Slices were maintained in a standard custom-made maintenance chamber gently and continuously aerated with carbogen gas for at least 60 min at room temperature (20–22°C) before being used for electrophysiology.

Slices were transferred to a submersion recording chamber and were continuously perfused at a rate of 2–4 ml/min with fully oxygenated aCSF at 32°C. Neurons were visualized using infra-red differential interference contract (IR-DIC) under an upright microscope (Olympus BXWI 51) equipped with a 40× objective (0.8 numerical aperture), an IR filter, DIC optics and a charge coupled device (CCD) video camera (Watac). For visualizing recorded neurons, 0.1% neurobiotin (Vectorlab) was added to all intercellular solutions.

Whole-cell patch-clamp recordings were performed with a Multiclamp 700B amplifier (Molecular Devices) and an Axopatch 200A amplifier (Molecular Devices). The signals were sampled at 20 kHz and low-pass filtered at 1 kHz. Series resistance (Rs) and input resistance (Rin) were frequently monitored throughout the experiments via a 10 mV, 250-ms hyperpolarizing step. Any large changes in holding current or noise characteristics were taken as early signs of cell loss and recordings were terminated. Experiments were also terminated if Rs exceeded 35 MΩ or if Rin changed >15% after break in the whole-cell mode. Rs (typical values of 10–30 MΩ) was compensated by 60–70% in the majority of the experiments. Membrane capacitance (Cm) was measured under voltage clamp at −50 mV using a hyperpolarizing 10 mV, 250-ms step. Cm was measured from the change in membrane charge taken from the integrated capacity transients (pClamp, Molecular Devices). All potentials cited here have not been corrected for liquid junction potentials (estimated using pClamp calculator as 9.2 mV). Slices were incubated in drug cocktails for minimum of 15 min before recording.

For evoked postsynaptic currents, a bipolar stimulating electrode (FHC) was placed 100–300 µm rostral to the recorded neuron and used to stimulate afferents at 0.03 Hz. Stimulus intensity was controlled using an ISO-flex stimulus isolator (AMPI) and adjusted to evoke monosynaptic events. Therefore, stimulation only elicited currents with a single peak, and fast rise and decay kinetics. GABA_A_ receptors were blocked by picrotoxin (100 µM). The whole-cell recording electrode (4–7 MΩ) was filled with an internal solution containing: 128 mM CsCH_3_SO_3_, 20 mM HEPES, 5 mM TEA-Cl, 2.8 mM NaCl, 0.4 mM EGTA, 2 mM MgATP, and 0.5 mM NaGTP (pH 7.25–7.35, 280–285 mOsm). The putative VTA dopamine neurons were initially voltage clamped at −70 mV and gradually shifted to +40 mV. Once a stable clamp was achieved, a single stimulus at an interval of 20 s was applied and eEPSCs were obtained. After at least 10 sweeps of stable current recording were successfully made, D-AP5 (50 µM) was applied to the slice for a minimum of 10 min, and pure AMPAR-mediated eEPSCs were recorded. NMDAR-mediated currents were obtained by a digital subtraction between the mixed current and the AMPAR current using Clampfit 10.2 (Molecular Devices). The AMPAR/NMDAR ratio was calculated by dividing the peak amplitude of the average AMPAR-mediated eEPSC by the peak amplitude of the NMDAR-mediated eEPSC.

For optogenetic and pharmacogenetic stimulation, VTA dopamine neurons were identified by the expression of mCherry or YFP. The whole-cell recording electrode (5–7 MΩ) was filled with an internal solution containing: 140 mM K-gluconate, 5 mM KCl, 10 mM HEPES, 0.1 mM EGTA, 2 mM MgCl_2_, 2 mM MgATP, and 0.2 mM NaGTP (pH 7.3–7.4, 280–285 mOsm). A blue light (470 nm) was delivered by TTL-control from a microscope-mounted LED to the entire field through the objective. After the achievement of stable current clamp, a rapid flash of light for 5 ms (25 Hz) with an interstimulus interval of 5 s was given for four times at the 60-s intersweep interval. The light intensity was adjusted according to the magnitude of its response. The yellow light (585 nm) was delivered by TTL-control from a microscope-mounted LED to the entire field through the objective. After the stable current clamp was achieved, +75-pA current step (12 s) was given, and two sets of 8-s continuous light stimulation were applied at 2-s intervals. The light intensity was adjusted according to the magnitude of its response. For the hM4Di experiments, 100 μM CNO (C0832, Sigma) was pipetted directly into the bath chamber after obtaining a stable spontaneous firing for 10 min. After the membrane potential was hyperpolarized, CNO was washed off with aCSF. For hM3Dq, 1 μM CNO was perfused onto the brain slice after obtaining a stable spontaneous firing for 10 min, and change of membrane potential and firing frequency was monitored for 20 min.

### In vivo electrophysiology

C57BL6 mice were anaesthetized with isofluorane and maintained with urethane during recording. Body temperature was maintained at 35 ± 0.5°C with a homeothermic heating blanket connected to a rectal thermometer (Harvard Apparatus). Hydration was maintained with injections of 0.45% saline or 0.9% saline every 3 h for mice in the salt depleted experiment and mice in the overnight fasted experiment. A craniotomy was performed above the VTA, removing a rectangular section of skull 3–3.6 mm from bregma, and 0.5 mm either side of the midline in length, avoiding damage to the underlying dura and mid-sagittal sinus. A glass electrode was then positioned ±0.4 mm mediolateral to bregma and within the range of −3.2 to −3.5 mm anterior-posterior to bregma. The glass electrode was then lowered at a speed of 10 µm/s to a depth of −3.5 mm from the dura. The electrode was then slowly lowered at 1 µm/s, stopping at spike detection. Extracellular recordings were made with glass microelectrodes (tip diameter, 1–1.5 μm; 15–25 MΩ) lowered into the VTA with a micromanipulator (single-axis IVM) controlled via LINLAB software and a PatchPad (all from Scientifica). Signals were AC-coupled, amplified (1000×) and bandpass filtered (0.3–5 kHz) with a Neurolog system (NL102G head-stage and DC preamplifier; Digitimer), and acquired on-line with a Micro1401 interface and SPIKE2 software (v6; Cambridge Electronic Design). Mains noise (50 Hz) was eliminated with “Humbug” filters (Quest Scientific). Electrophysiological recordings were collected from putative dopamine neurons (identified using electrophysiological criteria; Ungless and Grace, 2012) sampled using multiple penetrations within the VTA in a random order across an AP gradient of −3.2 to −3.5 mm in the left hemisphere and right hemisphere. Neurons were recorded once their baseline firing had stabilized, data were collected for a 3-min spike train. Five spike firing parameters were extracted and analyzed from *in vivo* recordings: firing rate, coefficient of variation (CV) of the interspike interval (ISI), spike wave form shape and duration from onset (defined as a change of >0.02 mV from baseline) to the negative trough (Ungless et al., 2004) and percentage of spikes within a burst (Grace and Bunney, 1984). Single-unit recordings were performed by an experimenter blind to condition. All parameters were analyzed with scripts and algorithms within Spike2 (CED).

### Experimental design and statistical analyses

Behavioral data were analyzed using a mixed ANOVA with time and concentration, where appropriate to analysis, as within subjects factors; and genotype (DATcre+ vs DATcre-) as a between subjects factor. An ANOVA was performed to test the consumed weight of the jellies, total beam breaks or preference score (this is the intake of one concentration jelly over the course of the session divided by total intake of the jellies in that session). Where significant interactions were observed, follow-up pairwise comparisons were conducted. Violations of sphericity were adjusted for using the Huynh–Feldt adjustment. Violations of normality were assessed by plotting the residuals of the data. All electrophysiological data were analyzed using non-parametric Mann–Whitney *U* tests. The significance level for all statistics was *p* < 0.05 (two-tailed).

## Results

### Optogenetic excitation of VTA dopamine neurons selectively decreases intake of high-concentration salt jellies during salt appetite

**Figure 1.**
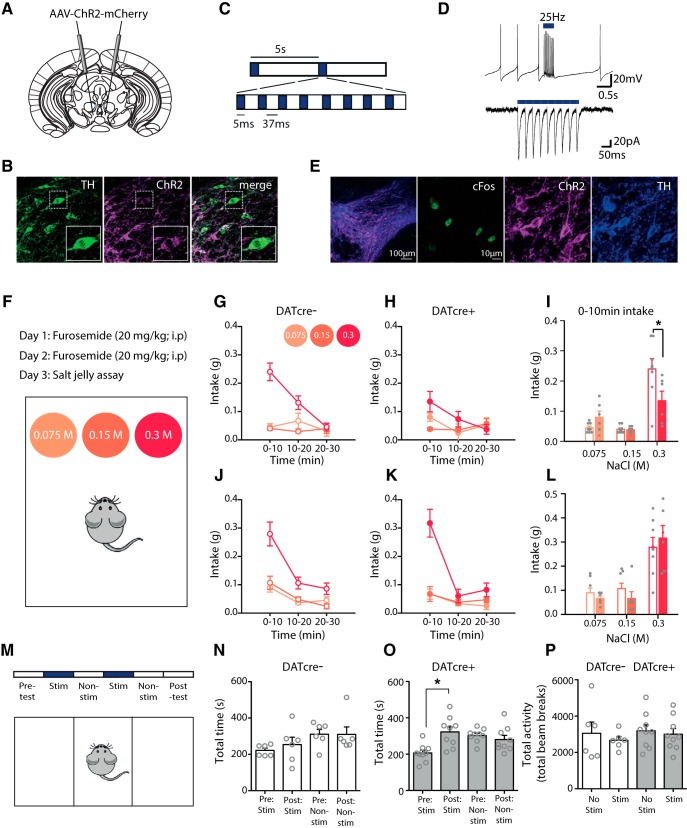
Optogenetic excitation of VTA dopamine neurons selectively decreases intake of high-concentration salt jellies during salt appetite. ***A***, DATcre- and DATcre+ mice were injected with a cre-dependent AAV carrying ChR2, conjugated to the fluorescent protein mCherry (AAV-ChR2-mCherry). ***B***, Colocalization of mCherry and TH confirmed expression of ChR2 in VTA dopamine neurons in DATcre+ mice. ***C***, Optical stimulation consisted of eight pulses: 5 ms on of a blue light laser, 37 ms off. ***D***, *Ex vivo* whole-cell patch recordings confirmed the optical blue light stimulation protocol was sufficient to depolarize the VTA TH+ positive cells leading to phasic bursts of activity. ***E***, DATcre+ (*n* = 4) and wild-type litter mates (*n* = 3) injected with AAV-ChR2-mCherry in the VTA were optogenetically stimulated using the same protocol. A significant increase in the number of dopamine neurons exhibiting cFos expression was observed in DATcre+ mice compared to DATcre- mice [23.1 ± 3.6 vs 12.9 ± 1.5; *t* = 2.326, *p* = 0.028; *N* = 16 and 12 (sections); immunostaining for tyrosine hydroxylase (TH) and cFos], confirming VTA dopamine neurons were activated in vivo by optical blue light stimulation. ***F***, An acute salt appetite was induced and assayed by injecting mice with the diuretic furosemide for 2 d before a preference test between three jellies of three different NaCl concentrations. DATcre- and DATcre+ mice were optogenetically stimulated with blue light during the preference test. DATcre- (*n* = 8; ***G***) and DATcre+ mice (*n* = 6; ***H***) differed in their intake of the different concentration salt jellies, time × genotype × concentration *F*_(4,48)_ = 3.6, *p* < 0.05. Main effects of time *F*_(2,24)_ = 10.3, *p* < 0.005, concentration *F*_(1.5,18.2)_ = 9.4, *p* < 0.005 and time × concentration *F*_(2.5,30.4)_ = 18.4, *p* < 0.001) were also revealed with statistical analysis. There was no significant interaction of time × genotype (*F*_(2,24)_ = 1.7, *p* > 0.1, N.S.). ***I***, A selective reduction in intake of the high-concentration salt jelly occurred during the first 10 min of the test session in the DATcre+ mice, concentration × genotype *F*_(2,24)_ = 7.1, *p* < 0.005, concentration *F*_(1.5,17.6)_ = 34.11, *p* < 0.001, pairwise comparisons 0.3 M salt DATcre- vs DATcre+ *p < 0.05. ***J***, ***K***, When tested a week later in the absence of stimulation, mice preferentially consumed the highest concentration salt jelly (concentration *F*_(1.3, 16.1)_ = 40.6, *p* < 0.001). Overall, mice decreased their consumption across time (*F*_(1.7,20.0)_ = 56.4, *p* < 0.001) presumably due to satiation which resulted in an overall change in preference for the high-concentration salt jelly (time × concentration *F*_(2.5,30.1)_ = 16.4, *p* < 0.001). No differences in intake were observed between salt-depleted groups across the session (time × genotype × concentration *F*_(4,48)_ = 1.7, *p* > 0.1, N.S., concentration × genotype, time × genotype, and genotype all *F* < 1, *p* > 0.5, N.S) (***L***) nor during the first 10 min of the session (genotype, concentration × genotype all *F* <1, *p* > 0.3). ***M***, DATcre- (n = 9) and DATcre+ (n = 6) mice injected with the cre-dependent AAV-ChR2-mcherry virus in the VTA and were tested using an biased CPP test. Preference was assessed for one of two chambers (pre-test) followed by optogenetic stimulation for 2 d in the non-preferred chamber (days 2 and 4) and 2 d of no stimulation in the preferred chamber (days 3 and 5). The last day (*post hoc* test), mice were tested for their preference in the absence of stimulation. ***N***, DATcre- mice (n = 6) showed no preference for the chamber where they had previously received stimulation (stimulation *F*_(1,5)_ = 2.9, *p* > 0.1, N.S.; session *F*_(1,5)_ = 2.6, *p* > 0.1, N.S.; session × stimulation *F* < 1, *p* > 0.7, N.S). ***O***, DATcre+ mice (n = 9) showed a significant preference for the chamber in which they had previously received stimulation (session × stimulation *F*_(1,8)_ = 7.0, *p* < 0.05), pairwise comparisons revealed this was specific to the stimulated chamber (pre- vs post-session *p < 0.01). ***P***, Optogenetic stimulation did not change locomotor activity as measured during the stimulation days of the unbiased CPP test (stimulation; stimulation × genotype; genotype all *F* < 1, *p* > 0.4, N.S.). Data represented as mean ± standard error of the mean (SEM).

To optogenetically excite VTA dopamine neurons, DATcre+ and DATcre- mice (Turiault et al., 2007) were stereotaxically injected with a cre-dependent AAV containing an EF1α promoter-driven ChR2 fused to mCherry (AAV-ChR2-mCherry; Fig. 1*A*). We observed strong mCherry expression, and colocalization with TH+ neurons, in VTA sections of DATcre+ mice (Fig. 1*B*). We then confirmed with *ex vivo* recordings that our stimulation protocol (Fig. 1*C*) excited identified VTA dopamine neurons (Fig. 1*D*). Furthermore, following photostimulation in awake behaving mice, we observed increased cFos expression selectively in dopamine neurons, providing evidence of *in vivo* activation (Fig. 1*E*).

We next investigated the effect of optogenetically exciting dopamine neurons on salt intake. Salt appetite was induced in mice by placing them on a low sodium diet and administering the sodium-wasting loop diuretic furosemide (Rowland et al., 2004; Fig. 1*F*). Salt intake was assessed using a three-choice salt jelly assay with three different concentrations [0.3, 0.15, and 0.075 M NaCl (Fig. 1*F*); no effect of genotype was observed on this assay in the absence of stimulation (data not shown)]. Optogenetic excitation of VTA dopamine neurons, initiated 5 min before exposure to salt jelly, selectively decreased consumption of the 0.3 M salt jelly in DATcre+ mice compared to DATcre- mice during the first 10 min of the test session, when salt intake was greatest (Fig. 1*G*–*I*). When mice were tested again one week later, in the absence of photo-stimulation, no group difference was observed, indicating that the effects of optogenetic stimulation were reversible (Fig. 1*J–L*).

One possible explanation of our results is that stimulation of VTA dopamine neurons induces an aversive state. Indeed, stimulation of mesocortical dopamine neurons (or dorsal raphe dopamine neurons) can induce a conditioned place aversion (Lammel et al., 2012; Gunaydin et al., 2014; Matthews et al., 2016). However, given the relatively lateral position of our laser fiber we considered it unlikely that we were stimulating mesocortical dopamine neurons. Nonetheless, we used a biased CPP test to assess the aversive properties of our optogenetic stimulation protocol. DATcre+ and DATcre- mice injected with AAV-ChR2 were first habituated to the three-compartment apparatus and their preferred compartment noted. The same optical stimulation procedure as used during the behavioral salt-jelly tests was then conducted in the non-preferred compartment for 2 d with alternating days in the preferred compartment without stimulation (Fig. 1*M*). Following this, mice were tested for their compartment preference in the absence of optical stimulation. We replicated the effect of Tsai et al. (2009) demonstrating that DATcre+ mice spent an increased amount of time in the compartment where they received stimulation versus time spent in this compartment before stimulation, whereas DATcre- mice spent a similar amount of time in the stimulation compartment before and after conditioning (Fig. 1*N*,*O*). This suggests that VTA dopamine neuron stimulation does not result in an aversive state.

The reduction in salt intake with optogenetic excitation of VTA dopamine neurons might be attributed to a general increase in locomotor activity competing with consumption (Carlton, 1963). However, analysis of locomotor activity during the four conditioning days revealed no differences in total activity between DATcre+ and DATcre- mice, with or without stimulation (Fig. 1*P*).

### Chemogenetic inhibition of VTA dopamine neurons does not affect intake of high-concentration salt jellies during salt appetite

**Figure 2.**
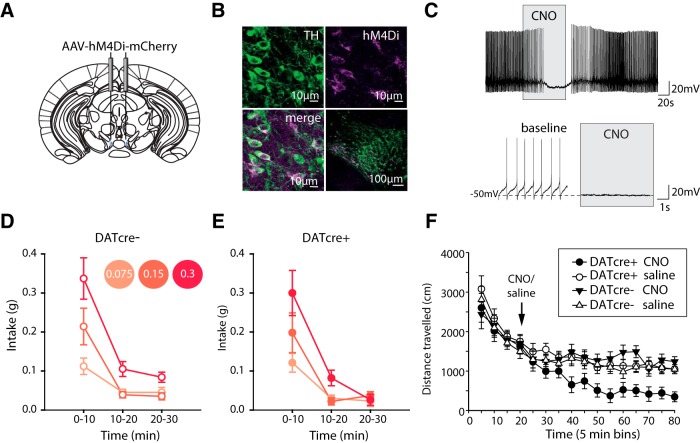
Chemogenetic inhibition of VTA dopamine neurons does not affect intake of high-concentration salt jellies during salt appetite. ***A***, DATcre+ mice (*n* = 9) and DATcre- mice (*n* = 11) were injected in the VTA with cre-dependent AAV carrying the Gi-coupled human M4 muscarinic DREADD coding sequence conjugated to the fluorescent protein mCherry (AAV-hM4Di-mCherry). ***B***, Colocalization of mCherry and TH staining confirmed expression of AAV-hM4Di-mCherry in VTA dopamine neurons of DATcre+ mice. ***C***, *Ex vivo* recordings confirmed CNO application to coronal VTA slices resulted in hyperpolarization of VTA TH+ve neurons in DATcre+ mice that had been previously injected with AAV-hM4Di-mCherry. ***D***, ***E***, Systemic CNO activation of AAV-hM4Di-mCherry before the salt appetite assay resulted in no significant effects on intake between genotypes (time × concentration × genotype, concentration × genotype, time × genotype all *F* < 1, all *p* > 0.5). Intake decreased over time (*F*_(1.3,23.9)_ = 100.3, *p* < 0.001) with preference in concentration also changing over time (*F*_(1.9,33.8)_ = 6.0, *p* < 0.001). ***F***, Systemic CNO activation of AAV-hM4Di-mCherry in the VTA of DATcre+ (*n* = 10) and DATcre- (*n* = 12) mice resulted in a significant reduction in locomotor activity in DATcre+ mice, treatment × genotype *F*_(1,18)_ = 8.5, *p* < 0.01 pairwise comparisons revealed significant effects only following CNO treatment, DATcre+ vs DATcre- *p* < 0.005. There was a significant interaction between treatment and time, with locomotor activity continuing to reduce with time in the CNO DATcre+ group (time × treatment *F*_(11,198)_ = 2.8, *p* < 0.005). However, this did not differ between genotypes (time × treatment × genotype *F*_(11,198)_ = 1.1, *p* > 0.3; time × genotype *F* < 1, *p* > 0.7). Data represented as mean ± SEM.

Next, we wanted to examine the effects of inhibition of VTA dopamine neurons on salt intake. To ensure robust inhibition of dopamine neurons we used a chemogenetic approach which tonically inhibits the spontaneous activity of neurons (Stachniak et al., 2014). A cre-dependent AAV containing the Gi-coupled human M4 muscarinic DREADD coding sequence (hM4Di; a G-protein coupled receptor that decreases cell excitability) conjugated to the fluorescent protein mCherry (AAV-hM4Di-mCherry), was injected into the VTA of DATcre+ and DATcre- mice (Fig. 2*A*). We observed strong mCherry expression, and colocalization with TH+ neurons, in VTA sections of DATcre+ mice (Fig. 2*B*). We then confirmed with *ex vivo* recordings that application of CNO inhibited action potential firing of identified VTA dopamine neurons (Fig. 2*C*). CNO was injected 30 min before testing, and had no effect on intake of jellies (Fig. 2*D*,*E*). As no obvious change in behavior was observed during the salt assay, we wanted to confirm we had an effective CNO dose to activate hM4Di *in vivo*. The same mice were placed in an open field chamber for 20 min, then injected with the same dose of CNO as used previously and returned to the chamber for 60 min. DATcre+ mice demonstrated significant decreases in their locomotor activity compared to DATcre- mice, suggesting that our chemogenetic approach was capable of inhibiting dopamine neurons (Fig. 2*F*). We conclude, therefore, that inhibiting dopamine neurons does not affect salt intake.

**Figure 3.**
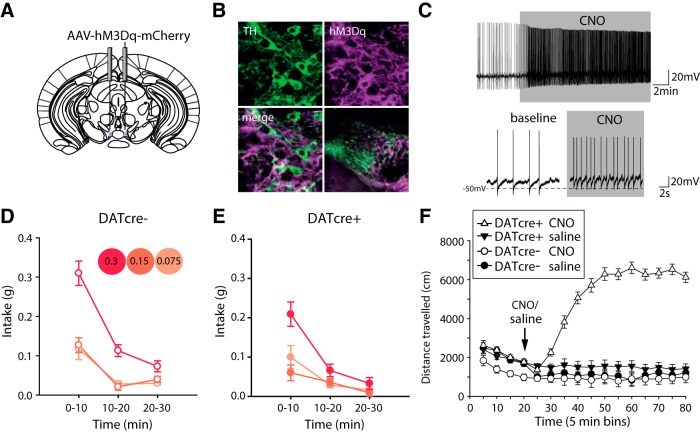
Chemogenetic excitation of VTA dopamine neurons induces behavioral hyperactivity and non-selective reduction in salt intake. ***A***, DATcre+ mice and DATcre- mice were injected with cre-dependent AAV carrying the Gq-coupled human M3 muscarinic DREADD coding sequence conjugated to the fluorescent protein mCherry (AAV- hM3Dq-mCherry). ***B***, Colocalization of mCherry and TH staining confirmed expression of AAV-hM4Di-mCherry in VTA dopamine neurons of DATcre+ mice. ***C***, *Ex vivo* recordings confirmed CNO application to coronal VTA slices resulted in depolarization of VTA TH+ve neurons in DATcre+ mice that had been previously injected with AAV-hM3Dq-mCherry. ***D***, ***E***, Systemic CNO activation of AAV-hM4Di-mCherry before the salt appetite assay resulted in a significant reduction in intake across the session of DATcre+ mice (*n* = 11) compared to DATcre- mice (*n* = 12), genotype × time *F*_(2,42)_ = 5.7, *p* < 0.01. Overall, intake decreased with time (*F*_(2,42)_ = 140.8, *p* < 0.001) and DATcre+ differ in intake to DATcre- (*F*_(1,21)_ = 16.5, *p* < 0.005). Unlike optogenetic excitation of VTA dopamine neurons, this difference in intake between DATcre+ and DATcre- mice was not due to changes in preference of concentration (time × concentration × genotype *F* < 1, *p* > 0.5, N.S.; concentration × genotype *F*_(2,42)_ = 2.1, *p* > 0.1, N.S; concentration *F*_(1.6,34.5)_ = 27.5, *p* < 0.001). Changes in preference of salt jelly changed overall over the course of the session regardless of the genotype of the mouse (time × concentration *F*_(3.1,64.4)_ = 10.4, *p* < 0.001). Significant differences in intake occurred primarily in the first and last thirds of the session, pairwise comparisons; *p* < 0.005 for intake during both 0–10 min and 20–30 min of the session for DATcre+ vs DATcre-. ***F***, DATcre+ (*n* = 15) and DATcre- mice (*n* = 12) infused with the same hM3Dq virus in the VTA and injected systemically with the same dose of CNO, significantly increased in locomotor behavior confirming the activation of the virus and effectiveness of the CNO dose genotype × drug *F*_(1,23)_ = 46.2, *p* < 0.001; drug *F*_(1,23)_ = 23.5, *p* < 0.001; genotype *F*_(1,23)_ = 71.1, *p* < 0.001. Data represented as mean ± SEM.

We also conducted complimentary chemogenetic experiments to excite dopamine neurons, by expressing hM3Dq in the VTA (Fig. 3*A–C*). However, this manipulation induced very high levels of locomotor activity (consistent with previous reports; Wang et al., 2013) confounding interpretation of the apparent reduction in intake seen across all salt jelly concentrations (Fig. 3*D–F*). This effect of chemogenetic excitation on locomotion, not seen with optogenetic excitation, may be due to the different temporal dynamics of the chemogenetic approach which is likely to have a slower onset and offset than the optogenetic stimulation, and/or the possibility that the chemogenetic approach excited a larger population of dopamine neurons compared to the more localized optogenetic approach.

### Optogenetic excitation of VTA dopamine neurons selectively decreases intake of high-concentration sucrose jellies following an overnight fast

**Figure 4.**
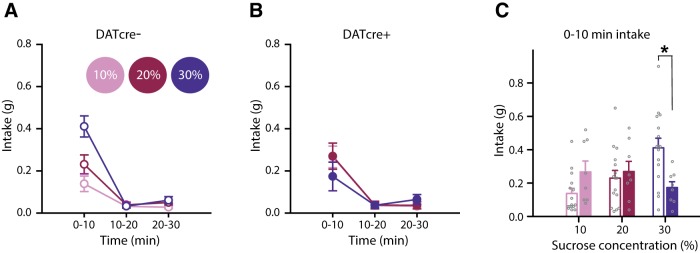
Optogenetic excitation of VTA dopamine neurons selectively decreases intake of high-concentration sucrose jellies following an overnight fast. ***A***, ***B***, Optogenetic excitation of VTA dopamine neurons resulted in a selective decrease in consumption of the high-concentration sucrose jelly in ***B*** DATcre+ mice (*n* = 8) with respect to ***A***, DATcre- mice (*n* = 15), time × concentration × genotype *F*_(4,84)_ = 5.2, *p* < 0.005, concentration × genotype *F*_(2,42)_ = 3.6, *p* < 0.05. There was no overall preference for one concentration (*F*_(2,42)_ = 1.6, *p* > 0.2), nor did a preference occur over time (time × concentration *F* < 1, *p* > 0.5), or overall intake differ between mice (time × genotype *F* < 1, *p* > 0.5; genotype *F* < 1, *p* > 0.4). The selective reduction in intake of the highest concentration sucrose jelly in DATcre+ (*n* = 8) mice with respect to DATcre- (*n* = 16) mice was specific to the first 10 min of the session, concentration × genotype *F*_(2,44)_ = 5.3, *p* < 0.01, pairwise comparisons 30% sucrose DATcre- vs DATcre+ *p < 0.01.

We next sought to test whether the effects of optogenetic excitation of VTA dopamine neurons generalized to other types of appetite, in particular appetite for sucrose following an overnight fast. DATcre+ and DATcre- mice were again injected with AAV-ChR2 in the VTA, implanted with optical fibers, and then allowed to recover for one week. Mice were then handled and habituated to the testing apparatus and sucrose jellies as in the salt experiment. At ∼4 P.M., the day before testing, home-cage chow was removed and mice were fasted overnight. The following day, optogenetic excitation of VTA dopamine neurons, which began 5 min before testing, selectively decreased consumption of the high-concentration sucrose jelly (assayed using three jellies of different sucrose concentrations: 10%, 20%, and 30%) in DATcre+ mice compared to DATcre- mice (Fig. 4*A*,*B*). This selective decrease in consumption of the high-concentration sucrose jelly in DATcre+ mice was observed during the first 10 min of the session when appetite was strongest (Fig. 4*C*). These results parallel those seen during salt appetite, suggesting that excitation of VTA dopamine neurons may have a general effect on appetites.

### Salt appetite and sucrose appetite do not affect firing activity or excitatory synaptic strength in dopamine neurons

**Figure 5.**
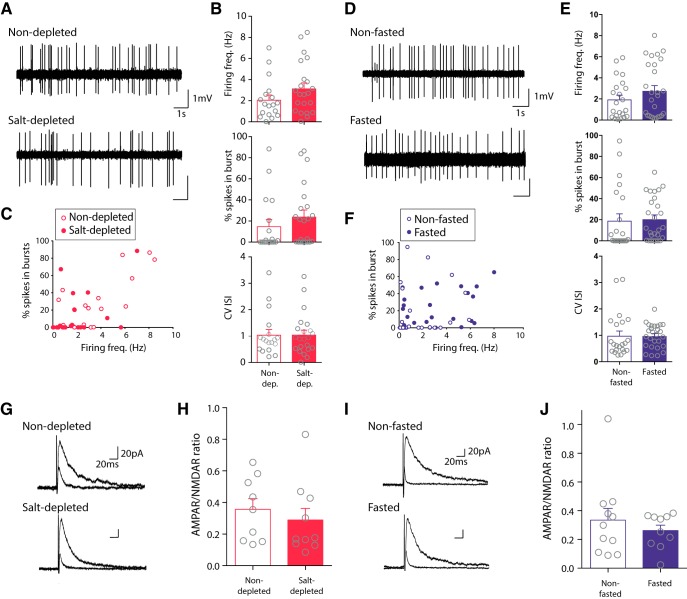
Salt appetite and sucrose appetite do not affect firing activity or excitatory synaptic strength in putative VTA dopamine neurons. ***A***, Traces of firing activity from putative dopamine neurons from C57BL6 mice either salt-depleted or non-depleted saline controls. ***B***, No differences in firing frequency of putative dopamine neurons were seen following salt depletion [*n* = 22(13)] with respect to control saline injected controls [*n* = 18(9); *U* = 135, *p* > 0.5, N.S.]. No differences in % spikes in bursts between groups (*U* = 148, *p* > 0.1, N.S.). The CV of the ISI did not differ between groups (*U* = 186, *p* > 0.7, N.S.). ***C***, Individual frequency of firing of each putative dopamine neuron against its % spikes in a burst. ***D***, Traces of firing activity from putative VTA dopamine neurons from C57BLk6 mice either fasted or non-fasted controls. ***E***, No differences in firing frequency of putative dopamine neurons were seen following fasting [*n* = 27(10)] with respect to controls [*n* = 22(10); *U* = 246, *p* > 0.3, N.S.]. No differences in % spikes in bursts between groups [*U* = 209, *p* > 0.8, N.S.]. The CV of the ISI did not differ between groups [*U* = 265, *p* > 0.5, N.S.]. ***F***, Individual frequency of firing of each putative dopamine neuron against its % spikes in a burst. ***G***, Example traces of excitatory postsynaptic potentials in putative dopamine neurons from salt-depleted and non-depleted mice. ***H***, AMPA/NMDA ratio was unaffected in dopamine neurons of salt-depleted [*n* = 10(5)] versus non-depleted mice [*n* = 9(4); *U* = 34, *p* > 0.1, N.S.]. ***I***, Example traces of excitatory postsynaptic potentials in putative dopamine neurons from fasted and non-fasted mice. ***J***, AMPAR/NMDAR ratio was unaffected in putative dopamine neurons of fasted [*n* = 10(5)] versus non-fasted mice [*n* = 11(6); *U* = 50, *p* > 0.1, N.S.]. Ns are cells (animals). Data represented as mean ± SEM.

Given the effect of increasing firing activity on salt and sucrose intake, we wanted to know what effect these appetites had on baseline dopamine neuron firing activity. Interestingly, despite the intensive study of the role of dopamine neurons in reward, little is known about how appetite affects their firing activity. One recent study reported an increase in burst firing, but not firing rate, of substantia nigra dopamine neurons in response to prolonged food restriction, but not an overnight fast (Branch et al., 2013). In addition, they observed an increase in excitatory synaptic strength following food restriction. We, therefore, examined *in vivo* firing activity and *ex vivo* excitatory synaptic strength in putative dopamine neurons in the VTA either after an overnight fast or during salt appetite. First, we conducted single-unit extracellular recordings of action potential activity from putative dopamine neurons in the VTA of anaesthetized mice. We observed no effect of either an overnight fast or salt appetite on firing frequency, burst activity, or firing regularity (Fig. 5*A–F*). Second, we conducted whole-cell recordings of synaptic currents in putative dopamine neurons in *ex vivo* acute brain slices. In particular, we assayed AMPAR/NMDAR ratios (a commonly used measure of synaptic strength in dopamine neurons; Ungless et al., 2001; Branch et al., 2013). We observed no effect of either an overnight fast or salt appetite on AMPAR/NMDAR ratios (Fig. 5*G–J*). Taken together, these results suggest that the acute induction of either a salt or sucrose appetite does not affect baseline firing activity, or excitatory synaptic strength, in VTA dopamine neurons. 

### Optogenetic excitation of VTA dopamine neurons does not disrupt salt concentration preference following an overnight fast

**Figure 6.**
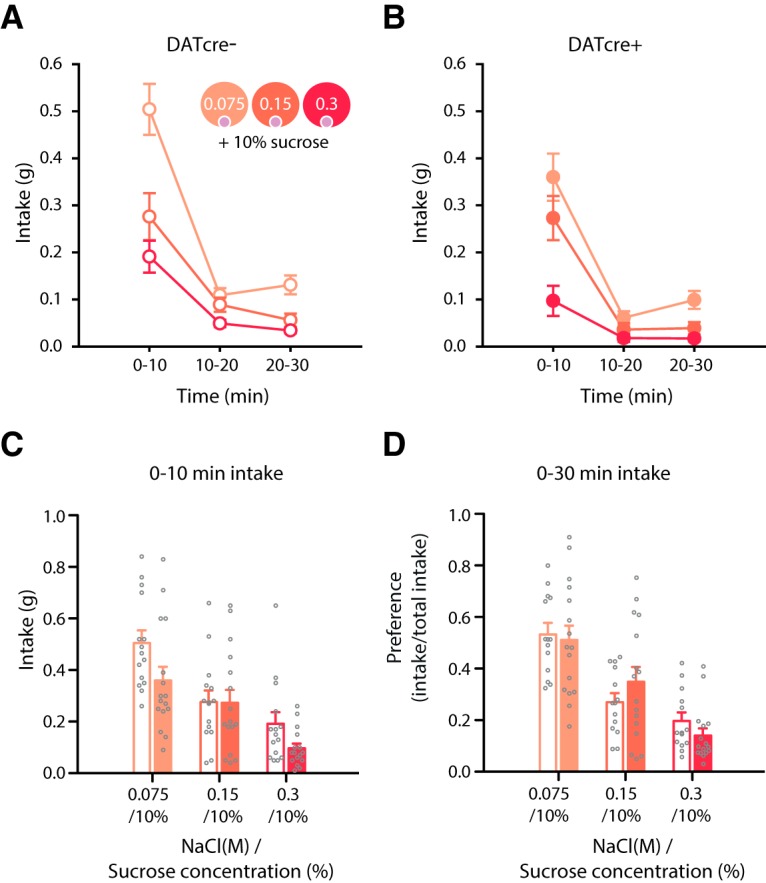
Optogenetic excitation of VTA dopamine neurons does not disrupt salt concentration preference following an overnight fast. ***A***, ***B***, Although optogenetic excitation of VTA dopamine neurons reduced overall intake in the DATcre+ (*n* = 16) compared to DATcre- mice (*n* = 14) following an overnight fast throughout the session, it did not affect salt concentration preference. Time × genotype × concentration *F*_(4112)_ = 1.1, *p* > 0.3, N.S. Overall intake was significantly reduced in DATcre+ mice (genotype *F*_(1,28)_ = 25.9, *p* < 0.001), which differed across time, time × genotype *F*_(2,56)_ = 3.6, *p* < 0.05. ***C***, DATcre+ and DATcre- mice preferentially consumed the low-concentration salt +10% sucrose jelly, concentration *F*_(1.7,48.8)_ = 21.3, *p* < 0.005 (pw comparisons-, intake was significantly different between all concentrations *p* < 0.05), which did not differ between genotypes, concentration × genotype *F* < 1, *p* > 0.5. Preference changed with time (time × concentration *F*_(2.4,68.2)_ = 10.3, *p* < 0.001). ***D***, Concentration preference did not differ between DATcre+ and DATcre- mice (concentration × genotype *F* < 1, *p* > 0.5) with all mice preferring the low salt concentration jelly (concentration *F*_(1.9,45.6)_ = 21.0, *p* < 0.001). Data represented as mean ± SEM.

Our results show that optogenetic excitation of VTA dopamine neurons selectively can reduce intake of both high-concentration salt and high-concentration sucrose. One possible interpretation of this effect is that the optogenetic excitation somehow leads to the inability to perceive differences in concentration or to exhibit preference behavior. To test this, following an overnight fast, we presented mice with three jellies of differing salt concentration, but the same concentration of sucrose (jelly 1: 0.075 M NaCl + 10% sucrose; jelly 2: 0.15 M NaCl +10% sucrose; jelly 3: 0.3 M NaCl + 10% sucrose) to assess whether mice could discriminate the differing concentrations of NaCl. DATcre+ mice and DATcre- mice exhibited a clear preference for the low-concentration salt jelly (0.075 M NaCl + 10% sucrose), as would be expected in this salt replete state (Fig. 6*A–C*). Importantly, as expected, optogenetic excitation of dopamine neurons reduced overall jelly intake in the DATcre+ mice, but there was no interaction with salt concentration. Furthermore, when intake was expressed as preference scores it was clear that there was no effect of optogenetic stimulation on preference (Fig. 6*D*).

## Discussion

Here, we showed that optogenetic excitation of VTA dopamine neurons specifically reduced intake of high-concentration salt during salt appetite. This effect was relatively rapid (i.e., occurring within minutes of stimulation) and reversible (i.e., it was not present a week later in the absence of excitation). We did not detect any aversive properties of the optogenetic excitation of VTA dopamine neurons, nor did it lead to an increase in locomotor activity. We also found that chemogenetic inhibition of dopamine neurons had no effect on salt-intake. Furthermore, we found that optogenetic excitation of VTA dopamine neurons also reduced intake of a high-concentration sucrose jelly following an overnight fast, complimenting recent reports of optogenetic excitation of VTA dopamine neurons inhibiting sucrose drinking (Mikhailova et al., 2016) and chemogenetic excitation of dopamine neurons inhibiting food intake (Boekhoudt et al., 2017). Taken together, these results suggest a general role of VTA dopamine neuron excitation in modulating intake during appetite. Importantly, we found that the specific reduction in intake of the high salt concentration jelly during salt appetite was not due to a disruption in the ability of the mice to demonstrate a preference. Although it is not possible for us to know which projection-specific populations of dopamine neurons we excited, it should be noted that because of the position of our optic fibers, it is likely that we preferentially stimulated dopamine neurons in more lateral parts of the VTA, which are more likely to project to the striatum, and avoided more medially located mesocortical dopamine neurons which can drive aversive behavior (Lammel et al., 2012). Consistent with this, we found that our stimulation protocol could generate a CPP, similar to that seen in previous studies (Tsai et al., 2009).

Despite the intensive study of the role of VTA dopamine neurons in reward processing, relatively little is known about how appetite affects their firing activity or synaptic properties. Our *in vivo* recordings in both salt-depleted and fasted mice revealed no effect of these appetite manipulations on spontaneous firing activity of putative VTA dopamine neurons. Consistent with this, an overnight fast does not change the spontaneous firing of substantia nigra dopamine neurons (Branch et al., 2013). However, more prolonged fasting or long-term food restriction has been shown to increase burst firing of dopamine neurons (Marinelli et al., 2006; Branch et al., 2013). Taken together these findings suggest that an acute appetite does not change dopamine neuron firing activity, but that more chronic manipulations of appetite may increase dopamine neuron firing activity possibly by engaging stress-related mechanisms. The excitatory inputs of midbrain dopamine neurons are highly sensitive to motivationally-significant events (e.g., a single exposure to addictive drugs such as cocaine, reward learning, stress, and long-term food restriction; Ungless et al., 2001; Saal et al., 2003; Stuber et al., 2008; Branch et al., 2013). We, therefore, tested whether manipulations of appetite used in this study changed synaptic strength in VTA dopamine neurons. No change was seen in AMPAR/NMDAR ratios following overnight fast or salt depletion, indicating that an acute appetite per se does not affect glutamatergic synaptic strength in dopamine neurons. We sampled from a broad population of putative dopamine neurons in the lateral parts of the VTA with unknown projection targets, and it is therefore possible that our sample contained some mesocortical dopamine neurons which do not exhibit synaptic plasticity to appetitive events (Lammel et al., 2011).

Our finding that optogenetic excitation of VTA dopamine neurons resulted in a reduction in intake during appetite is consistent with reports of suppressed intake with the systemic administration of either D-amphetamine (Kraeuchi et al., 1985), cocaine (Wellman et al., 2002), or dopamine receptor agonists (Cincotta et al., 1997, Kuo, 2002; Chen et al., 2008). A number of studies have attributed the anorexic effects of amphetamine to its modulation of the dopaminergic system. Supporting this, dopamine antagonists (Leibowitz, 1975; Garattini et al., 1976), electrolytic lesions and 6-OHDA lesions of the nigrostriatal pathway (Fibiger et al., 1973; Carey and Goodall, 1975), and 6-OHDA lesions of the neostriatum (Joyce and Iversen, 1984) all alleviate the hypophagic effects of amphetamine. Direct evidence of the role of dopamine in amphetamine-induced hypophagia is found in dopamine-deficient (DD) mice that are insensitive to the hypophagic effects of amphetamine (Cannon et al., 2004). Moreover, viral restoration of dopamine to the caudate putamen of these mice reinstated amphetamine-induced hypophagia implicating dopamine signaling within the dorsal striatum in this phenomenon (Cannon et al., 2004). The specificity of these effects to the dopamine system within this region is supported by the failure to ameliorate amphetamine-induced hypophagia using a variety of manipulations targeting alternative neurochemical systems thought to be altered by amphetamine administration (Cannon et al., 2004; Sotak et al., 2005). Furthermore, no effect was found with viral restoration of dopamine signaling in the nucleus accumbens, which is consistent with a large body of literature on the failure to disrupt the primary motivational properties of food with lesions to the nucleus accumbens or dopamine antagonism within this region (Roberts et al., 1977; Caine and Koob, 1994; Salamone et al., 2005).

A reduction in intake with increased dopamine activity may appear counterintuitive considering dopamine’s role in behavioral activation (Robbins and Everitt, 2007; Salamone et al., 2009; Syed et al., 2016) and food-seeking behavior (Wise, 2006). However, it has been proposed that optimal levels of dopaminergic activity are essential for the activation of motivationally-relevant behavior (Heffner et al., 1977; Palmiter, 2007; Robbins, 2010). The activation of motivated behavior is dependent on an inverted u-shaped function of dopamine activity. In the case of appetite, optimal levels of dopamine activity result in food-seeking behavior (Roitman et al., 2004). However, too little dopamine, exemplified in DD mice, inhibits feeding as these mice die of starvation unless maintained with daily injections of L-DOPA (Zhou and Palmiter, 1995; Szczypka et al., 1999). Too much dopamine, as may be the case with the present optogenetic study and previous studies (Alnaser and Cooper, 1994; van der Hoek and Cooper, 1994; Scislowski et al., 1999; Mikhailova et al., 2016), also results in inhibition of intake during appetite. Our optogenetic excitation protocol may, therefore, have resulted in dopamine activity beyond the optimal levels for engaging in food consumption during a state of appetite. Importantly, the use of pharmacological manipulations often produces confounding results to those observed with more rapid optogenetic manipulations (Otchy et al., 2015). Our findings, therefore, usefully build on these previous pharmacological manipulations by showing that direct excitation of dopamine neurons can relatively rapidly suppress intake. Lastly, our observation that chemogenetic inhibition had no effect on intake is reminiscent of the failure to affect food consumption with 6-OHDA lesion and dopamine antagonism of the striatum (Aberman and Salamone, 1999; Baldo et al., 2002).

We propose that optogenetic excitation of VTA dopamine neurons reduces salt or sucrose appetite such that mice are no longer driven to consume a high-concentration of a relevant reinforcer. Alternative theoretical accounts of these results could be that increased tonic dopamine levels switch behavior from exploitation of a current food resource to exploration of the environment for alternative food resources (Cohen et al., 2007; Beeler et al., 2010, 2012; Humphries et al., 2012) perhaps by increasing behavioral vigor (Niv et al., 2007). Although it is difficult to separate the different contributions of phasic versus tonic dopamine in our task, and the effects of our stimulation protocol on them, this account of our results seems unlikely for several reasons. First, optogenetic excitation of VTA dopamine neurons during the stimulation days of the CPP test showed no difference in locomotor activity compared to non-stimulated sessions. Second, intake was decreased throughout the 30 min intake test. If stimulation had led to more exploration, but no suppression of appetite, then we might have expected mice to eventually return to consume the jellies. Another possibility we addressed is that the stimulation procedure disrupted the ability of the mice to demonstrate preferential consumption of one jelly. However, when fasted mice were presented with three jellies with differing concentrations of salt, but the same concentration of sucrose, optogenetic excitation of VTA dopamine neurons did not affect preferential consumption of the low-concentration salt jelly. This suggests the ability to exhibit preference behavior is unaffected by optogenetic stimulation of dopamine neurons and is consistent with the observation that hyperdopaminergic mutant mice exhibit normal hedonic “liking” responses to sweet tastes (Pecina et al., 2003).

The reduction in intake of both salt and sucrose with optogenetic excitation of VTA dopamine neurons suggests a common mechanism may have been disrupted for both nutrient rewards. Dopamine within the dorsal striatum has been shown to play a specific role in feeding behavior as viral restoration of dopamine signaling in dopamine deficient mice rescues feeding behavior (Cannon et al., 2004). Our optical fiber was positioned preferentially above the dorsolateral VTA. Considering the topography of projections of the VTA, it is possible that we excited neurons projecting to more dorsal regions of the striatum. Activation of dopamine projections from the dorsolateral VTA to more dorsal regions of the striatum may provide a nutritional signal despite a state of hunger such that mice are no longer motivated to consume the highest concentration jelly.

## Synthesis

Reviewing Editor: Carmen Sandi, Swiss Federal Institute of Technology Decisions are customarily a result of the Reviewing Editor and the peer reviewers coming together and discussing their recommendations until a consensus is reached. When revisions are invited, a fact-based synthesis statement explaining their decision and outlining what is needed to prepare a revision will be listed below. The following reviewer(s) agreed to reveal their identity: Stephanie Borgland, Ronald Keiflin

This manuscript addresses the hypothesis that phasic dopaminergic neuronal stimulation reduces motivated salt intake. They find that while food preference is intact (mice preferred sucrose with the lowest salt concentration), the mice reduce consumption and preference for a motivationally relevant food (salt in salt-deprived state, or sucrose in food deprived state), when dopamine neurons are phasically opto-activated during a consumption session. The authors suggest that because food preference are intact and animals continue to prefer contexts associated with phasic stimulation, that phasic stimulation of dopamine neurons is biologically relevant for appetite suppression. This confirms and extends previous work showing that increased activity in VTA-DA neurons reduces consumption of other types of reward (food, ethanol).

The authors have addressed most of the reviewers' previous comments. Still there are a few further minor issues to be addressed, particularly in the Discussion, regarding interpretation of data and clarification of hypotheses and conclusions:

- Discussion: Please, consider adding another plausible interpretation: that hedonic responses to sucrose are not modulated by elevated dopamine concentration (see Pecina et al 2003 J Neurosci 23(28):9395-402). Accordingly, it is somewhat predictable that preferences would be intact regardless of phasic dopaminergic stimulation.

- Discussion: Regarding the interpretation of your Gq-DREADD data, please consider adding that it is also likely that a larger population of dopamine neurons is stimulated with CNO administration as opposed to optogenetic stimulation (potentially leading to the enhanced locomotor activity observed).

- Discussion: Regarding your text: “The artificial phasic activation of VTA dopamine neurons with optogenetics may result in phasic signals of reward that update the overall reward rate reported by tonic dopamine. As such, mice are no longer motivated to consume jellies during appetite.” It would be helpful to explain how these two sentences are connected. Indeed, it is not immediately obvious how the second sentence is a logical consequence of the first one. Niv et al. 2007 (to which authors refer to) suggest that tonic dopamine reports reward rate, with elevated dopamine tone (high reward rate) increasing the response vigor/motivation. Although “motivation” refers here primarily to reward-seeking behaviors rather than consumption, this model nonetheless seems at odds with the authors conclusions that increased phasic dopamine reduces motivation to consume food.

- Discussion: The authors argue that this overall reduction of consumption (via elevated dopamine tone) is somewhat independent of the effects that phasic dopamine stimulation has on outcome preference. If this is indeed the hypothesis, maybe the authors could elaborate or at least make this point more explicit in the text -perhaps acknowledge that it is difficult to tease apart the contribution of phasic vs. tonic dopamine levels in this task.

- Line 96, line 99 references are not in parentheses

- Add product numbers for the antibodies used
